# (2*Z*)-2-(4-Methyl­phen­yl)-3-(2-naphth­yl)prop-2-enenitrile

**DOI:** 10.1107/S1600536809034229

**Published:** 2009-09-09

**Authors:** Abdullah Mohamed Asiri, Mehmet Akkurt, Islam Ullah Khan, Muhammad N. Arshad, Salman A. Khan

**Affiliations:** a Chemistry Department, Faculty of Science, King Abdul-Aziz University, PO Box 80203, Jeddah 21589, Saudi Arabia; b Department of Physics, Faculty of Arts and Sciences, Erciyes University, 38039 Kayseri, Turkey; c Department of Chemistry, Government College University, Lahore, Pakistan

## Abstract

In the title compound, C_20_H_15_N, the dihedral angle between the naphthalene and benzene rings is 60.30 (16)°. The crystal packing features very weak inter­molecular C—H⋯π inter­actions.

## Related literature

For background on the commercial importance and applications of styryl dyes, see: Haidekker *et al.* (2001 ▶); Hamer (1964 ▶); Li *et al.* (1998 ▶); Makoto *et al.* (2000*a* ▶,*b* ▶); Mousnier *et al.* (2004 ▶); Park *et al.* (2001 ▶); Pommeret *et al.* (1995 ▶); Spalletti (2004 ▶). For reference structural data, see: Allen *et al.* (1987 ▶).
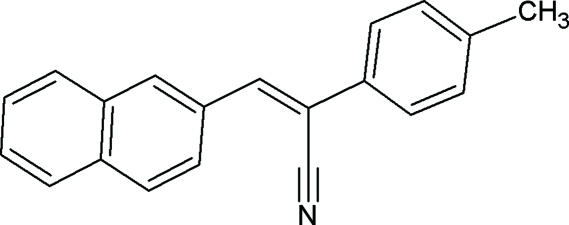


## Experimental

### 

#### Crystal data

C_20_H_15_N
*M* *_r_* = 269.33
Orthorhombic, 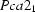

*a* = 12.3194 (11) Å
*b* = 16.4796 (16) Å
*c* = 7.2596 (7) Å
*V* = 1473.8 (2) Å^3^
*Z* = 4
Mo *K*α radiation
μ = 0.07 mm^−1^
*T* = 296 K
0.37 × 0.28 × 0.13 mm

#### Data collection

Bruker Kappa APEXII CCD diffractometer
Absorption correction: none
16248 measured reflections
3659 independent reflections
1549 reflections with *I* > 2σ(*I*)
*R*_int_ = 0.092

#### Refinement

*R*[*F* ^2^ > 2σ(*F* ^2^)] = 0.058
*wR*(*F* ^2^) = 0.174
*S* = 0.95
3659 reflections
192 parameters
1 restraint
H-atom parameters constrained
Δρ_max_ = 0.21 e Å^−3^
Δρ_min_ = −0.21 e Å^−3^

### 

Data collection: *APEX2* (Bruker, 2007 ▶); cell refinement: *SAINT* (Bruker, 2007 ▶); data reduction: *SAINT*; program(s) used to solve structure: *SHELXS97* (Sheldrick, 2008 ▶); program(s) used to refine structure: *SHELXL97* (Sheldrick, 2008 ▶); molecular graphics: *ORTEP-3 for Windows* (Farrugia, 1997 ▶); software used to prepare material for publication: *WinGX* (Farrugia, 1999 ▶) and *PLATON* (Spek, 2009 ▶).

## Supplementary Material

Crystal structure: contains datablocks global, I. DOI: 10.1107/S1600536809034229/hb5070sup1.cif

Structure factors: contains datablocks I. DOI: 10.1107/S1600536809034229/hb5070Isup2.hkl

Additional supplementary materials:  crystallographic information; 3D view; checkCIF report

## Figures and Tables

**Table 1:** Hydrogen-bond geometry (Å, °)

*D*—H⋯*A*	*D*—H	H⋯*A*	*D*⋯*A*	*D*—H⋯*A*
C2—H2⋯*Cg*1^i^	0.93	2.98	3.682 (4)	134
C7—H7⋯*Cg*2^ii^	0.93	2.93	3.695 (4)	140
C9—H9⋯*Cg*1^ii^	0.93	2.78	3.473 (4)	133

Symmetry codes: (i) 
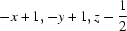
; (ii) 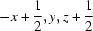. *Cg*1 and *Cg*2 are the centroids of the C1–C3/C8–C10 and C3–C8 rings, respectively.

**Fig. 1.**
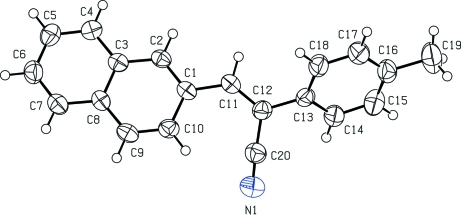
View of the molecular structure of (I): displacement ellipsoids for non-H atoms are drawn at the 50% probability level.

**Fig. 2.**
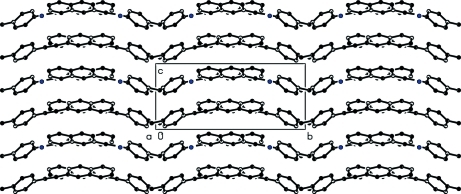
The packing of (I) viewed down the a axis. H atoms have been omitted for clarity.

## supplementary crystallographic information

### Comment

Styryl dyes are of commercial importance, not only because of their applications as pigment, but also due to their high technology applications, such as sensitizers formerly (Hamer, 1964), electroluminescence (Makoto *et al.*, 2000a,b), photochromism (Spalletti, 2004), photography (Li *et al.*, 1998), fluorescent probes (Haidekker *et al.*, 2001), optical recording materials (Park *et al.*, 2001), laser dyes (Pommeret *et al.*, 1995) and in the field of medication (Mousnier *et al.*, 2004).

The structure of the title compound (I) is shown in Fig. 1. All bond lengths (Allen *et al.*, 1987) and bond angles in (I) may be regarded as normal. The naphthalene ring in (I) is almost planar, with the maximum deviations of -0.008 (3), 0.015 (3), -0.009 (4) and -0.008 (4) Å for atoms C1, C2, C5 and C8, respectively. The mean plane of the naphthalene ring makes a dihedral angle of 60.30 (16)° with the benzene ring of the 2-(4-methylphenyl) group.

The crystal packing is stabilized by weak intermolecular C—H···π interactions between the centroids of the two six-membered rings (C1–C3/C8–C10) and (C3–C8) of naphthalene of the adjacent molecules (Table 1). Fig. 2 shows the packing arrangement in the unit cell, as viewed down the *a* axis.

### Experimental

A mixture of 4-methyl benzylcyanide (1.00 g, 0.0076 mol) and 2-napthaldehyde (1.18 g, 0.0076 mol) in anhydrous ethanol (15 ml), in the presence of pyridine was refluxed at 353 K for 3 h with continuous stirring. Progress of reaction was monitored by TLC. After completion of the reaction solution was cooled. The heavy precipitate thus obtained was collected by filtration and purified by recrystallization from methanol and chloroform [m.p.: 412 K, yield: 57%] to yield pale yellow prisms of (I).

### Refinement

H atoms were placed in calculated positions, with C—H = 0.93 and 0.96 Å, and refined using a riding model, with *U*_iso_(H)= 1.5*U*_eq_(C) for methyl and 1.2*U*_eq_(C) for others; the methyl were allowed to rotate but not to tip.  The absolute structure parameter was indeterminate.

### Crystal data

**Table:**

C_20_H_15_N	*F*(000) = 568
*M**_r_* = 269.33	*D*_x_ = 1.214 Mg m^−^^3^
Orthorhombic, *P**c**a*2_1_	Mo *K*α radiation, λ = 0.71073 Å
Hall symbol: P 2c -2ac	Cell parameters from 1202 reflections
*a* = 12.3194 (11) Å	θ = 3.3–19.2°
*b* = 16.4796 (16) Å	µ = 0.07 mm^−^^1^
*c* = 7.2596 (7) Å	*T* = 296 K
*V* = 1473.8 (2) Å^3^	Prism, pale yellow
*Z* = 4	0.37 × 0.28 × 0.13 mm

### Data collection

**Table:**

Bruker Kappa APEXII CCD diffractometer	1549 reflections with *I* > 2σ(*I*)
Radiation source: sealed tube	*R*_int_ = 0.092
graphite	θ_max_ = 28.3°, θ_min_ = 1.2°
φ and ω scans	*h* = −16→16
16248 measured reflections	*k* = −21→21
3659 independent reflections	*l* = −9→9

### Refinement

**Table:**

Refinement on *F*^2^	Primary atom site location: structure-invariant direct methods
Least-squares matrix: full	Secondary atom site location: difference Fourier map
*R*[*F*^2^ > 2σ(*F*^2^)] = 0.058	Hydrogen site location: inferred from neighbouring sites
*wR*(*F*^2^) = 0.174	H-atom parameters constrained
*S* = 0.95	*w* = 1/[σ^2^(*F*_o_^2^) + (0.0732*P*)^2^] where *P* = (*F*_o_^2^ + 2*F*_c_^2^)/3
3659 reflections	(Δ/σ)_max_ < 0.001
192 parameters	Δρ_max_ = 0.21 e Å^−^^3^
1 restraint	Δρ_min_ = −0.21 e Å^−^^3^

### Special details

**Table:**

Geometry. Bond distances, angles *etc*. have been calculated using the rounded fractional coordinates. All su's are estimated from the variances of the (full) variance-covariance matrix. The cell e.s.d.'s are taken into account in the estimation of distances, angles and torsion angles
Refinement. Refinement on *F*^2^ for ALL reflections except those flagged by the user for potential systematic errors. Weighted *R*-factors *wR* and all goodnesses of fit *S* are based on *F*^2^, conventional *R*-factors *R* are based on *F*, with *F* set to zero for negative *F*^2^. The observed criterion of *F*^2^ > σ(*F*^2^) is used only for calculating -*R*-factor-obs *etc*. and is not relevant to the choice of reflections for refinement. *R*-factors based on *F*^2^ are statistically about twice as large as those based on *F*, and *R*-factors based on ALL data will be even larger.

### Fractional atomic coordinates and isotropic or equivalent isotropic displacement parameters (Å^2^)

**Table:**

	*x*	*y*	*z*	*U*_iso_*/*U*_eq_	
N1	0.2102 (3)	0.2374 (2)	0.2270 (6)	0.0755 (18)	
C1	0.4123 (2)	0.4123 (2)	0.3160 (5)	0.0387 (11)	
C2	0.4702 (2)	0.4813 (2)	0.2783 (5)	0.0384 (11)	
C3	0.4291 (3)	0.5598 (2)	0.3140 (5)	0.0371 (11)	
C4	0.4872 (3)	0.6316 (2)	0.2717 (5)	0.0459 (14)	
C5	0.4449 (3)	0.7059 (2)	0.3090 (5)	0.0510 (14)	
C6	0.3431 (3)	0.7129 (2)	0.3916 (6)	0.0537 (14)	
C7	0.2845 (3)	0.6454 (2)	0.4337 (5)	0.0477 (16)	
C8	0.3250 (3)	0.5669 (2)	0.3945 (5)	0.0374 (11)	
C9	0.2669 (3)	0.4948 (2)	0.4371 (5)	0.0448 (14)	
C10	0.3079 (3)	0.4205 (2)	0.3993 (6)	0.0426 (11)	
C11	0.4613 (2)	0.3337 (2)	0.2763 (5)	0.0424 (11)	
C12	0.4168 (2)	0.2602 (2)	0.2486 (5)	0.0420 (11)	
C13	0.4813 (3)	0.1865 (2)	0.2136 (5)	0.0422 (14)	
C14	0.4457 (3)	0.1283 (2)	0.0903 (6)	0.0543 (14)	
C15	0.5086 (3)	0.0608 (2)	0.0502 (7)	0.0633 (17)	
C16	0.6068 (3)	0.0487 (2)	0.1335 (6)	0.0550 (16)	
C17	0.6417 (3)	0.1063 (2)	0.2582 (7)	0.0620 (18)	
C18	0.5807 (3)	0.1741 (2)	0.2980 (6)	0.0543 (14)	
C19	0.6730 (4)	−0.0271 (2)	0.0951 (8)	0.0840 (19)	
C20	0.3006 (3)	0.2492 (2)	0.2411 (7)	0.0503 (14)	
H2	0.53910	0.47630	0.22730	0.0460*	
H4	0.55540	0.62770	0.21750	0.0550*	
H5	0.48410	0.75230	0.27940	0.0610*	
H6	0.31510	0.76400	0.41800	0.0640*	
H7	0.21680	0.65090	0.48910	0.0570*	
H9	0.19910	0.49870	0.49250	0.0540*	
H10	0.26780	0.37440	0.42780	0.0510*	
H11	0.53670	0.33460	0.26870	0.0510*	
H14	0.37860	0.13450	0.03350	0.0650*	
H15	0.48350	0.02310	−0.03500	0.0750*	
H17	0.70810	0.09910	0.31690	0.0750*	
H18	0.60650	0.21190	0.38240	0.0650*	
H19A	0.64500	−0.07140	0.16670	0.1260*	
H19B	0.74750	−0.01760	0.12780	0.1260*	
H19C	0.66850	−0.04030	−0.03350	0.1260*	

### Atomic displacement parameters (Å^2^)

**Table:**

	*U*^11^	*U*^22^	*U*^33^	*U*^12^	*U*^13^	*U*^23^
N1	0.0474 (19)	0.072 (3)	0.107 (4)	−0.0020 (18)	0.000 (2)	−0.009 (2)
C1	0.0332 (17)	0.047 (2)	0.036 (2)	0.0056 (17)	0.0002 (16)	−0.0009 (19)
C2	0.0332 (17)	0.048 (2)	0.034 (2)	0.0018 (16)	−0.0001 (16)	−0.0039 (18)
C3	0.0353 (17)	0.048 (2)	0.028 (2)	0.0005 (16)	−0.0054 (16)	−0.0042 (17)
C4	0.0437 (19)	0.047 (2)	0.047 (3)	−0.0043 (18)	−0.001 (2)	0.000 (2)
C5	0.058 (2)	0.044 (2)	0.051 (3)	−0.005 (2)	−0.006 (2)	−0.004 (2)
C6	0.056 (2)	0.045 (2)	0.060 (3)	0.006 (2)	−0.005 (2)	−0.011 (2)
C7	0.042 (2)	0.054 (3)	0.047 (3)	0.0066 (19)	−0.0003 (18)	−0.007 (2)
C8	0.0392 (19)	0.044 (2)	0.029 (2)	0.0072 (17)	−0.0026 (16)	−0.0033 (18)
C9	0.0374 (19)	0.058 (2)	0.039 (3)	0.0041 (19)	0.0064 (18)	0.001 (2)
C10	0.0418 (19)	0.046 (2)	0.040 (2)	−0.0020 (17)	0.0063 (17)	0.0063 (19)
C11	0.0363 (17)	0.046 (2)	0.045 (2)	0.0053 (17)	0.0031 (17)	−0.0007 (19)
C12	0.0411 (18)	0.041 (2)	0.044 (2)	−0.0011 (17)	−0.0014 (19)	0.0056 (19)
C13	0.0407 (19)	0.036 (2)	0.050 (3)	−0.0034 (16)	−0.0008 (18)	0.0054 (18)
C14	0.049 (2)	0.047 (2)	0.067 (3)	0.002 (2)	−0.007 (2)	−0.006 (2)
C15	0.076 (3)	0.047 (3)	0.067 (3)	0.002 (2)	−0.007 (3)	−0.005 (2)
C16	0.063 (3)	0.041 (2)	0.061 (3)	0.007 (2)	0.014 (2)	0.008 (2)
C17	0.051 (2)	0.053 (3)	0.082 (4)	0.005 (2)	−0.010 (2)	0.000 (3)
C18	0.054 (2)	0.044 (2)	0.065 (3)	0.0016 (19)	−0.010 (2)	−0.008 (2)
C19	0.102 (3)	0.058 (3)	0.092 (4)	0.024 (3)	0.017 (3)	−0.006 (3)
C20	0.047 (2)	0.051 (2)	0.053 (3)	0.001 (2)	0.005 (2)	0.002 (2)

### Geometric parameters (Å, °)

**Table:**

N1—C20	1.135 (5)	C15—C16	1.367 (6)
C1—C2	1.370 (4)	C16—C17	1.380 (6)
C1—C10	1.428 (5)	C16—C19	1.518 (5)
C1—C11	1.458 (5)	C17—C18	1.377 (5)
C2—C3	1.413 (5)	C2—H2	0.9300
C3—C4	1.417 (5)	C4—H4	0.9300
C3—C8	1.414 (5)	C5—H5	0.9300
C4—C5	1.358 (5)	C6—H6	0.9300
C5—C6	1.395 (5)	C7—H7	0.9300
C6—C7	1.361 (5)	C9—H9	0.9300
C7—C8	1.415 (5)	C10—H10	0.9300
C8—C9	1.421 (5)	C11—H11	0.9300
C9—C10	1.353 (5)	C14—H14	0.9300
C11—C12	1.345 (5)	C15—H15	0.9300
C12—C13	1.474 (5)	C17—H17	0.9300
C12—C20	1.444 (4)	C18—H18	0.9300
C13—C14	1.383 (5)	C19—H19A	0.9600
C13—C18	1.384 (5)	C19—H19B	0.9600
C14—C15	1.387 (5)	C19—H19C	0.9600
			
N1···H10	2.7800	H2···C8^iv^	3.0200
N1···H4^i^	2.9300	H4···H2	2.5000
N1···H5^i^	2.8200	H4···C1^iv^	3.0100
N1···H19A^ii^	2.8800	H4···C10^iv^	2.9700
C5···C13^iii^	3.549 (5)	H4···N1^vii^	2.9300
C6···C13^iii^	3.591 (5)	H5···N1^vii^	2.8200
C10···C20	3.049 (5)	H7···H9	2.5200
C13···C5^iv^	3.549 (5)	H9···H7	2.5200
C13···C6^iv^	3.591 (5)	H9···C1^viii^	3.0700
C20···C10	3.049 (5)	H9···C2^viii^	2.9600
C1···H9^v^	3.0700	H9···C3^viii^	2.9900
C1···H4^iii^	3.0100	H10···N1	2.7800
C2···H9^v^	2.9600	H10···C12	2.9300
C3···H9^v^	2.9900	H10···C20	2.5000
C3···H2^iii^	3.0800	H11···C18	2.7100
C8···H2^iii^	3.0200	H11···H2	2.3500
C10···H4^iii^	2.9700	H11···H18	2.3500
C11···H18	2.8000	H14···C20	2.6000
C12···H10	2.9300	H15···H19C	2.5100
C14···H19B^ii^	3.0600	H15···C16^ix^	2.9000
C16···H15^vi^	2.9000	H15···C17^ix^	3.0300
C17···H15^vi^	3.0300	H17···H19B	2.4100
C18···H11	2.7100	H18···C11	2.8000
C20···H10	2.5000	H18···H11	2.3500
C20···H14	2.6000	H19A···N1^x^	2.8800
H2···H4	2.5000	H19B···H17	2.4100
H2···H11	2.3500	H19B···C14^x^	3.0600
H2···C3^iv^	3.0800	H19C···H15	2.5100
			
C2—C1—C10	118.4 (3)	C1—C2—H2	119.00
C2—C1—C11	118.8 (2)	C3—C2—H2	119.00
C10—C1—C11	122.7 (3)	C3—C4—H4	119.00
C1—C2—C3	122.5 (3)	C5—C4—H4	120.00
C2—C3—C4	123.0 (3)	C4—C5—H5	120.00
C2—C3—C8	118.5 (3)	C6—C5—H5	120.00
C4—C3—C8	118.6 (3)	C5—C6—H6	120.00
C3—C4—C5	121.1 (3)	C7—C6—H6	120.00
C4—C5—C6	120.4 (3)	C6—C7—H7	120.00
C5—C6—C7	120.4 (3)	C8—C7—H7	119.00
C6—C7—C8	121.0 (3)	C8—C9—H9	119.00
C3—C8—C7	118.6 (3)	C10—C9—H9	119.00
C3—C8—C9	118.5 (3)	C1—C10—H10	120.00
C7—C8—C9	122.9 (3)	C9—C10—H10	120.00
C8—C9—C10	121.6 (3)	C1—C11—H11	114.00
C1—C10—C9	120.5 (3)	C12—C11—H11	114.00
C1—C11—C12	131.4 (2)	C13—C14—H14	120.00
C11—C12—C13	123.3 (3)	C15—C14—H14	119.00
C11—C12—C20	121.5 (3)	C14—C15—H15	119.00
C13—C12—C20	115.1 (3)	C16—C15—H15	119.00
C12—C13—C14	120.8 (3)	C16—C17—H17	119.00
C12—C13—C18	121.5 (3)	C18—C17—H17	119.00
C14—C13—C18	117.7 (3)	C13—C18—H18	120.00
C13—C14—C15	121.0 (4)	C17—C18—H18	120.00
C14—C15—C16	121.2 (4)	C16—C19—H19A	109.00
C15—C16—C17	117.7 (3)	C16—C19—H19B	110.00
C15—C16—C19	121.0 (4)	C16—C19—H19C	110.00
C17—C16—C19	121.3 (4)	H19A—C19—H19B	109.00
C16—C17—C18	121.7 (4)	H19A—C19—H19C	109.00
C13—C18—C17	120.6 (4)	H19B—C19—H19C	109.00
N1—C20—C12	176.0 (5)		
			
C10—C1—C2—C3	−1.8 (5)	C3—C8—C9—C10	−1.3 (6)
C11—C1—C2—C3	−179.5 (3)	C7—C8—C9—C10	−179.5 (4)
C2—C1—C10—C9	1.0 (6)	C8—C9—C10—C1	0.5 (6)
C11—C1—C10—C9	178.5 (4)	C1—C11—C12—C13	−178.6 (4)
C2—C1—C11—C12	−158.4 (4)	C1—C11—C12—C20	4.9 (7)
C10—C1—C11—C12	24.1 (6)	C11—C12—C13—C14	−142.5 (4)
C1—C2—C3—C4	−178.5 (3)	C11—C12—C13—C18	35.5 (6)
C1—C2—C3—C8	1.1 (5)	C20—C12—C13—C14	34.2 (5)
C2—C3—C4—C5	−179.7 (4)	C20—C12—C13—C18	−147.8 (4)
C8—C3—C4—C5	0.7 (5)	C12—C13—C14—C15	176.8 (4)
C2—C3—C8—C7	178.8 (3)	C18—C13—C14—C15	−1.3 (6)
C2—C3—C8—C9	0.5 (5)	C12—C13—C18—C17	−177.5 (4)
C4—C3—C8—C7	−1.6 (5)	C14—C13—C18—C17	0.6 (6)
C4—C3—C8—C9	−179.9 (3)	C13—C14—C15—C16	1.2 (6)
C3—C4—C5—C6	0.5 (6)	C14—C15—C16—C17	−0.4 (6)
C4—C5—C6—C7	−0.8 (6)	C14—C15—C16—C19	177.4 (4)
C5—C6—C7—C8	−0.2 (6)	C15—C16—C17—C18	−0.3 (6)
C6—C7—C8—C3	1.4 (6)	C19—C16—C17—C18	−178.1 (4)
C6—C7—C8—C9	179.6 (4)	C16—C17—C18—C13	0.2 (6)

Symmetry codes: (i) *x*−1/2, −*y*+1, *z*; (ii) *x*−1/2, −*y*, *z*; (iii) −*x*+1, −*y*+1, *z*+1/2; (iv) −*x*+1, −*y*+1, *z*−1/2; (v) −*x*+1/2, *y*, *z*−1/2; (vi) −*x*+1, −*y*, *z*+1/2; (vii) *x*+1/2, −*y*+1, *z*; (viii) −*x*+1/2, *y*, *z*+1/2; (ix) −*x*+1, −*y*, *z*−1/2; (x) *x*+1/2, −*y*, *z*.

### Hydrogen-bond geometry (Å, °)

**Table:**

*D*—H···*A*	*D*—H	H···*A*	*D*···*A*	*D*—H···*A*
C2—H2···Cg1^iv^	0.93	2.98	3.682 (4)	134
C7—H7···Cg2^viii^	0.93	2.93	3.695 (4)	140
C9—H9···Cg1^viii^	0.93	2.78	3.473 (4)	133

Symmetry codes: (iv) −*x*+1, −*y*+1, *z*−1/2; (viii) −*x*+1/2, *y*, *z*+1/2.
